# Identification and isolation of high avidity tumor-specific CD8 T cells by experimentally assessing pMHC-TCR binding parameters with soluble pMHC complexes

**DOI:** 10.1186/2051-1426-3-S2-P263

**Published:** 2015-11-04

**Authors:** Julien Schmidt, Michael Hebeisen, Philippe Guillaume, Morgane Magnin, Nathalie Rufer, Immanuel Luescher

**Affiliations:** 1 Ludwig Center for Cancer Research, Epalinges, Switzerland; 2 Department of Oncology, Epalinges, Switzerland; 3 TCMetrix Ltd, Epalinges, Switzerland

## 

Adoptive transfer of high avidity tumor-specific CD8 T cells is a promising treatment for late stage cancers. Because most tumor antigens are self-antigens, tumor-specific T cells typically express low affinity T cell antigen receptors (TCR) due to thymic negative selection, which accounts mostly for their poor tumoricidal activity. To identify rare, most tumor-reactive T cells we developed a robust and accurate screening assay. The functional avidity of T cell responses is related to pMHC-TCR binding parameters, with an assumed optimal range of pMHC-TCR affinity for the most potent interaction. We recently described the development of NTAmers[1], reversible pMHC multimers built on switchable Ni^2+^-NTA-His-tag complexes, that allow accurate measurement of monomeric pMHC-TCR dissociation kinetics on living tumor-specific CD8 T cells by flow cytometry. We found a good correlation between off-rates and cellular responses on cloned CD8 T cells from cancer patients[2]. Moreover we developed pMHC dimers allowing measurement of pMHC-TCR association and dissociation kinetics on cancer-specific T cells. Combined measurement of pMHC-TCR on and off rates allowed accurate identification and isolation of functional high avidity tumor-specific CD8 T cells. In vivo experiments confirmed that high avidity CD8 T cells isolated from a cancer patient conferred superior tumor control. This novel technology has further allowed us to describe the functional involvement of the CD8 co-receptor in this interaction. Using CD8-binding deficient pMHC complexes, we observed that CD8 increases on CD8 T cells the off-rate by 3-4 fold but has no discernable effect on the on-rate.

In conclusion, NTAmers and pMHC dimers enable precise measurement of pMHC-TCR binding parameters under physiological conditions and subsequent isolation of live functionally active tumor-specific CD8 T cells. Translation of these techniques to the single cell level for high-throughput screening and isolation of rare and highly functional tumor-specific CD8 T cells directly from cancer patients is in progress.
